# Deciphering aging-associated molecular mechanisms in bone marrow derived hematopoietic stem cells in the elderly using NGS data

**DOI:** 10.6026/973206300200180

**Published:** 2024-02-29

**Authors:** Hind A Alkhatabi

**Affiliations:** 1 Department of Biochemistry, College of Science, University of Jeddah, Jeddah, Saudi Arabia

**Keywords:** Aging, hematopoietic stem cells, bone marrow, GREIN, BioJupies, RNA sequencing, gene ontologies, Next Generation Knowledge Discovery, L1000CDS2, drugs, natural products

## Abstract

Aging is a complex process that is not yet fully understood. Despite advancements in research, a deeper understanding of the underlying biological mechanisms is necessary to develop interventions that promote healthy longevity. The aim of this study was to elucidate the complex mechanisms associated with healthy aging and longevity in healthy elderly individuals. The RNA sequencing (RNA-seq) data used in this study was obtained from the Gene Expression Omnibus (GEO) database (accession number GSE104406), which was collected from Fluorescent Activated Cell Sorting (FACS) of human bone marrow derived human hematopoietic stem cells (BM-HSCs) (Lineage-, CD34+, CD38-) young (18-30 years old) and aged (65-75 years old) donors who had no known hematological malignancy, with 10 biological replicates per group. The GEO RNA-seq Experiments Interactive Navigator (GREIN) software was used to obtain raw gene-level counts and filtered metadata for this dataset. Next generation knowledge discovery (NGKD) tools provided by BioJupies were used to obtain differentially regulated pathways, gene ontologies (GO), and gene signatures in the BM-HSCs. Finally, the L1000 Characteristic Direction Signature Search Engine (L1000CDS2) tool was used to identify specific drugs that reverse aging-associated gene signatures in old but healthy individuals. The down-regulation of signaling pathways such as longevity regulation, proteasome, Notch, apoptosis, nuclear factor kappa B (NFkB), and peroxisome proliferator-activated receptors (PPAR) signaling pathways in the BM-HSCs of healthy elderly. GO functions related to negative regulation of bone morphogenetic protein (BMP), telomeric DNA binding, nucleoside binding, calcium -dependent protein binding, chromatin-DNA binding, SMAD binding, and demethylase activity were significantly downregulated in the BM-HSCs of the elderly compared to the healthy young group. Importantly, potential drugs such as salermide, celestrol, cercosporin, dorsomorphin dihydrochloride, and LDN-193189 monohydrochloride that can reverse the aging-associated signatures in HSCs from healthy elderly were identified. The analysis of RNA-seq data based on NGKD techniques revealed a plethora of differentially regulated pathways, gene ontologies, and drugs with anti-aging potential to promote healthspan in the elderly.

## Background:

Aging is a complex process influenced by genetic, epigenetic, and environmental factors [1] and is a primary risk factor for various chronic conditions, including cancer, cardiovascular diseases, diabetes, and neurodegenerative disorders. [2] The global population of older individuals is increasing rapidly due to the rise in human lifespan, with an estimated 2.1 billion people aged over 60 years by 2050, accounting for 80% of older individuals in low- and middle-income countries. [3] [4] Despite the increase in the average life expectancy, which is projected to be 72.3 years by 2050, the intricate process of aging remains poorly understood. [3] [4] Understanding the biology of aging and the complexities associated with it can lead to the development of methods to extend lifespan and reduce the incidence of age-related diseases. [5] To explore the intrinsic processes of aging, a multidisciplinary approach has been proposed that encompasses critical areas, such as DNA damage and repair, stem cell function, cellular senescence, and biological pathways. [6] By targeting these areas, researchers may be able to develop treatments that enhance healthy aging, also known as the healthspan. [4] [6] Hematopoietic stem cells (HSCs) play a vital role in the continual production of all mature blood cell types throughout an individual's lifetime and self-renew and differentiate to maintain and replenish the blood cell population. [7] As people age, HSC function declines, leading to hematopoietic aging, which is characterized by a reduced self-renewal capacity, decreased differentiation potential, and increased susceptibility to stress. [7] [8] The aging process at the HSCs has been linked to an increased incidence of hematological disorders as well as a decline in adaptive immunity and a heightened risk of developing myeloid malignancies, including myelodysplastic syndromes (MDS) and acute myeloid leukemia (AML). [9,10, 11,12,13] To better understand the mechanisms of hematopoietic aging and to develop strategies to maintain HSC function and promote healthy aging, it is essential to investigate gene expression patterns and identify molecular alterations associated with cellular and molecular processes using next-generation sequencing (NGS) technology. In the current study, RNA sequencing (RNA-seq) data for bone marrow-derived hematopoietic stem cells (BM-HSCs) from healthy young and elderly individuals were retrieved from the Gene Expression Omnibus (GEO) database and analyzed using state-of-the-art next-generation knowledge discovery (NGKD) tools. Therefore, it is of interest to identify the pathways, gene ontologies (GOs), and potential drugs that can reverse the aging-related gene signatures present in BM-HSCs of elderly individuals, thereby promoting healthy longevity or healthspan.

## Methodology:

## Ethical Statement:

This study relies on RNA-seq datasets obtained from the Gene Expression Omnibus (GEO) [14] [15], thereby obviating the need for Institutional Review Board (IRB) approval, as no animals or humans was involved in the research.

## Data Source:

RNA-seq data from the Gene Expression Omnibus (GEO) database (accession number GSE104406) was utilized in the present study. This data was collected from Fluorescent Activated Cell Sorting (FACS) isolated human bone marrow derived human HSCe (Lineage-, CD34+, CD38-) (BM-HSCs) young (18-30) and aged (65-75) donors who had no known hematological malignancy, with 10 biological replicates per group. [16] GEO RNA-seq Experiments Interactive Navigator (GREIN) software was used to obtain raw gene-level counts and filtered metadata for the dataset, as described previously.[17]

## BioJupies Analysis:

The BioJupies platform offers analysis plug-ins for 14 RNA-seq libraries that automatically generate, store, and deploy Jupyter Notebooks containing the outcomes of RNA-seq data analyses. [18] In order to detect differentially expressed genes (DEGs) between healthy young and healthy elderly persons, the raw counts were transformed into log10 counts per million (log CPM) using the limma R package. [19] The Jupyter Notebooks produced for RNA-seq analysis encompass the complete analysis pipeline, with method descriptions, enrichment analysis, interactive data visualizations, and additional components. [18] The principal component analysis (PCA) function from the sklearn Python library was used to perform PCA, and the log CPM values were transformed into Z-scores. The x-axis of the volcano plot displays the log2 fold changes of DEGs, while the y-axis displays the corrected and transformed (-log10) p-values, obtained based on the Benjamini-Hochberg method. [20] [21]

## WebGestalt Analysis:

The analysis of DEGs obtained from healthy elderly individuals were further clarified with a fold change cut-off of 1.5 and P value ≤ 0.05 and the WEB-based GEne SeT AnaLysis Toolkit (WebGestalt) (wGSEA) analysis was performed as previously described [22] For each type of analysis, Gene Ontology (GO) terms, including molecular function (GO-MF), and biological process (GO-BP), were selected and for pathway analysis KEGG and Reactome databases were selected. The reference list for each analysis comprised all mapped gene symbols from the chosen platform genome, Homo sapiens as species, and the parameters for the enrichment analysis set at a minimum of 5 and a maximum of 2000 IDs in the category, a false discovery rate (FDR) of P ≤ 0.05, computed using the Benjamini-Hochberg (BH) method, and a significance level of the top 10, as previously described. [23]

## L1000CDS2 Analysis:

The ultra-fast L1000 Characteristic Direction Signature Search Engine (L1000CDS2) method [24], was used to decipher the top 25 drugs and natural products that may counteract aging-related gene signatures in BM-HSCs of elderly individuals.

## Results:

**Figure 1:**
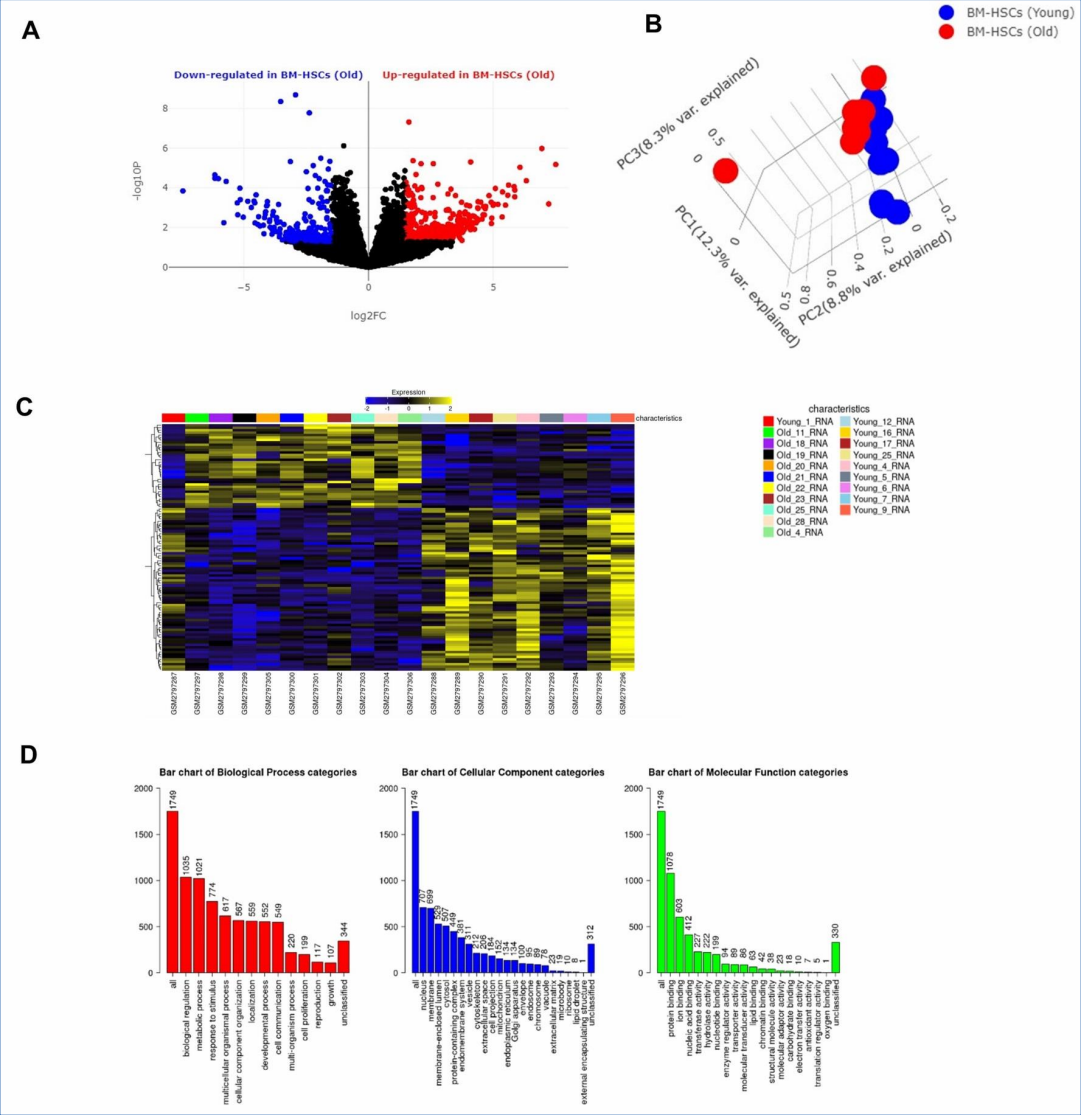
(A) Volcano plot of the DEGs derived from the bone marrow derived hematopoietic stem cells (BM-HSCs) of healthy old vs healthy young comparison using BioJupies tool. (B) Principal component analysis (PCA) of the study group based on differentially expressed genes (DEGs) using BioJupies tool. (C) Heatmap across comparison samples based on the top 100 DEGs derived from the characteristics (healthy old vs healthy young) ranked by adjusted p values based on GREIN.(D) GO Slim Summary of the DEGs used in WebGestalt analysis for biological process (Red), cellular component (Blue), and molecular function (Green).

In the present study, an extensive analysis of next-generation sequencing data from BM-HSCs of healthy old and young individuals to gain insights into the complex mechanisms involved in aging was conducted. Utilizing the filtered metadata and raw counts from GREIN, 15028 DEGs were identified between the two groups (aged, healthy vs. young, healthy as depicted in the volcano plot (Figure 1A derived from BioJupies analysis. PCA of the study groups (Figure 1B) showed a distinct separation of the healthy old and healthy young groups. Additionally, a heatmap was generated (Figure 1C) depicting the top 100 DEGs ranked by adjusted p-values (Figure 1C). GO Slim Summary of the filtered 1807 DEGs were obtained from WebGestalt Analysis and were depicted as bar graphs for biological process (Red), cellular component (Blue), and molecular function (Green) (Figure 1D).

**Figure 2:**
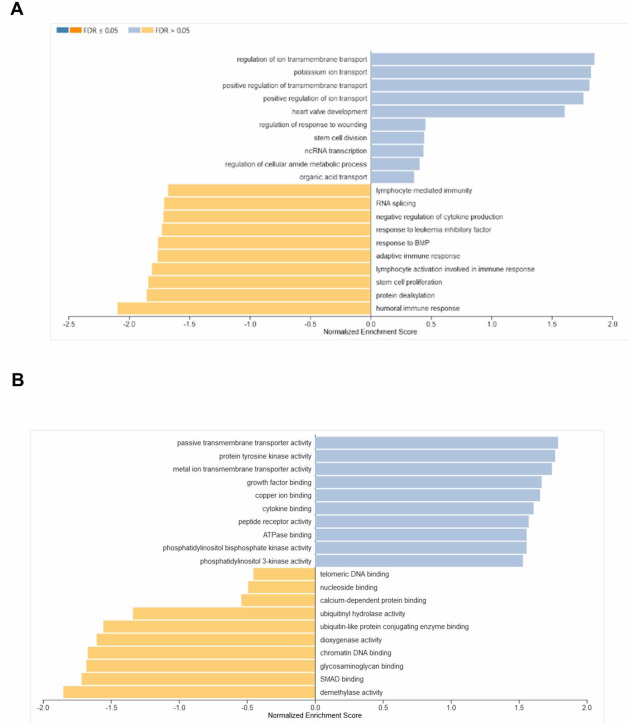
Differentially regulated Pathways in the BM-HSCs of healthy old compared to healthy young individuals. (A) The bar shows the results of the gene set enrichment analysis for KEGG and (B) Reactome pathways in the BM-HSCs of healthy old compared to healthy young individuals.

DEGs were further studied using the WebGestalt tool to identify pathways that were differentially regulated in the BM-HSCs of elderly compared to healthy young individuals. Kyoto Encyclopedia of Genes and Genomes (KEGG) pathways, including NF-kappa B signaling, longevity regulation, Peroxisome Proliferator-Activated Receptors (PPAR) signaling, cholesterol metabolism, Notch signaling, proteasome and other signaling mechanisms were negatively enriched (P < 0.05) in healthy elderly compared with healthy young individuals. The results of the gene set enrichment analysis are illustrated in the bar graph, which displays the normalized enrichment score and false discovery rate (FDR) as indicated by the intensity of the color in the labels (Figure 2A). Furthermore, Reactome pathway analysis of DEGs revealed the negative enrichment of a variety of pathways such as activation of NF-kappa B in B cells, regulation of apoptosis, Vpu mediated degradation of CD4, ubiquitination-dependent degradation of Cyclin D and Cyclin D1, degradation of AXIN, and dishevelled (DVL) and other signaling pathways in the BM-HSCs of elderly individuals (Figure 2B).

**Figure 3:**
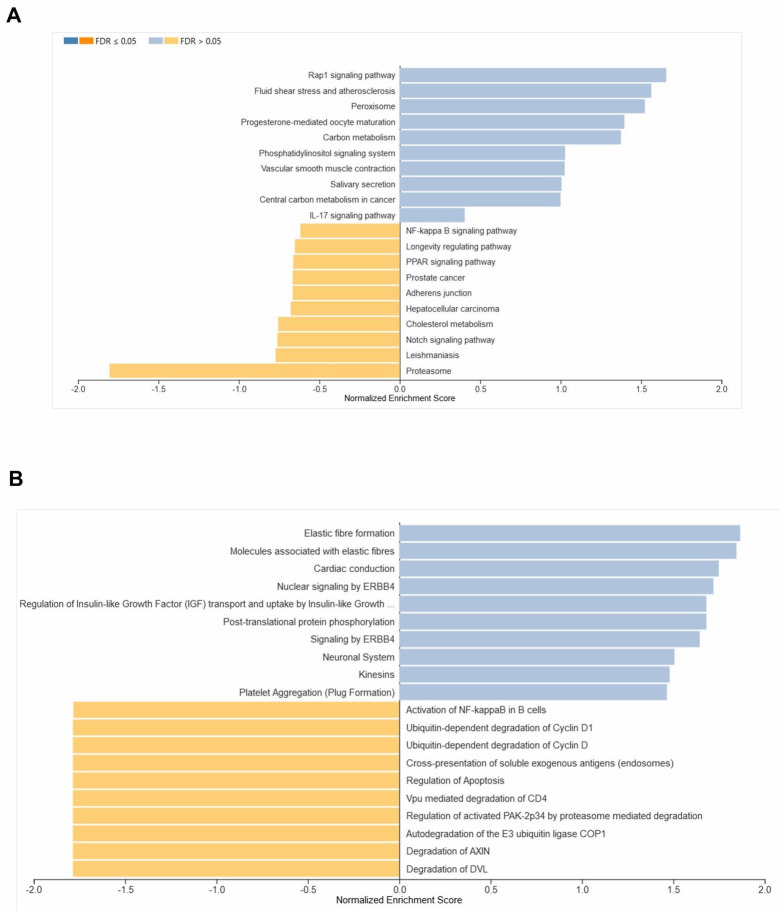
Differentially regulated Gene Ontologies (GO) in the BM-HSCs of healthy old compared to healthy young individuals. (A) The bar shows the results of the gene set enrichment analysis for Biological Process and (B) Molecular function in the BM-HSCs of healthy old compared to healthy young individuals.

**Figure 4:**
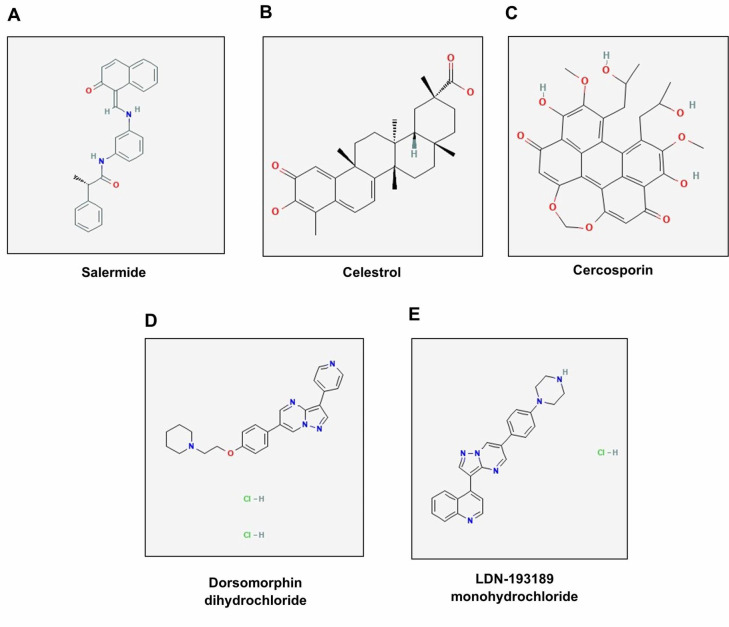
Potential drugs or natural products such as (A) Salermide (B) Celestrol (C) Cercosporin (D) Dorsomorphin dichloride, and (E) LDN-193189 mono-hydrochloride that reverse DEGs in the BM-HSCs of healthy elderly individuals compared with healthy young individuals based on L1000CDS2 analysis.

Besides, the differentially regulated GO-BP and GO-MF in the BM-HSCs of healthy old compared to healthy young individuals were identified using the WebGestalt tool. The negative enrichment of GO-BPs such as lymphocyte-mediated immunity, RNA splicing, negative regulation of cytokine production, response to leukemia inhibitory factor, response to Bone Morphogenetic Protein (BMP), adaptive immune response, lymphocyte activation involved in immune response, stem cell proliferation, protein deacetylation, and humoral immune response in BM-HSCs of healthy old when compared to healthy young were observed as depicted in Figure 3A. Moreover, the GO-MFs such as telomeric DNA binding, nucleoside binding, calcium-dependent protein binding, ubiquitinyl hydrolase activity, ubiquitin-like protein conjugating enzyme-binding, dioxygenase activity, chromatin DNA binding, glycosaminoglycan binding, SMAD binding, and demethylase activity were negatively enriched (P<0.05) in the BM-HSCs of healthy old compared to healthy young individuals (Figure 3B). In this study, L1000CDS2 tool was used to identify the top 25 drugs or natural products that reverse DEGs in the BM-HSCs of healthy elderly individuals. The results revealed potential drugs or natural products, such as Salermide, Celestrol, Cercosporin dichloride, Dorsomorphin monohydrochloride, and LDN-193189 monohydrochloride (Figure 4), which reverse DEGs in the BM-HSCs of healthy elderly individuals compared to healthy young individuals (Table 1).

## Discussion:

In this study, a thorough examination of the alterations in BM-HSCs that occur with age in healthy elderly individuals was conducted using RNA-seq data and cutting-edge NGKD techniques as previously described [15] [25]. The current study aimed to uncover differentially regulated pathways and GOs potentially linked with aging in the BM-HSCs of healthy elderly individuals. Furthermore, the research aimed to uncover potential therapeutic interventions capable of reversing the aging associated intricate mechanisms and promoting healthy longevity or healthspan. The dataset GSE104406 was obtained using the GREIN platform, which provided RNA-seq data (raw counts) along with associated metadata. The quality control, expression pattern visualization, sample size analysis, and power analysis were performed using the GREIN platform (Data not shown - check with author), as previously described. [17] The DEGs obtained from BioJupies analysis [18] were then filtered with a 1.5-fold cutoff and a p-value of ≤ 0.05, along with corresponding metadata, and used as input files for the WebGestalt analysis. [22]

In this study, a negative enrichment of genes linked to the NF-kappa B signaling pathway, which is crucial for the regulation of aging, was observed in BM-HSCs in healthy elderly individuals. [1] NF-κB acts as a central factor where various signaling pathways converge. Signals that promote aging activate NF-κB, while signals that promote longevity inhibit its activation. [1] [26] This could partly explain the negative enrichment of the longevity regulating pathway in BM-HSCs of healthy elderly individuals. On the contrary, the PPARs, NOTCH, and Proteasome signaling pathways were found to be downregulated in the BM-HSCs of healthy elderly. PPARs are a group of nuclear receptors that regulate genes involved in fat metabolism, inflammation, and cell proliferation. [27] Studies have demonstrated that activating PPARs, particularly PPARα and PPARγ, can enhance critical aspects of aging, such as extending lifespan in animal models. [27] Activation of PPARs is believed to promote healthy cellular function, reduce inflammation, and improve mitochondrial function, all of which may contribute to healthy aging. [27] The Notch signaling pathway is a cell-to-cell communication system that plays a vital role in development, cell fate determination, and stem cell function. As we age, Notch signaling can become dysregulated, leading to issues with tissue regeneration and stem cell function. Some research suggests that activating Notch signaling in specific contexts may help to improve tissue repair and promote healthy aging, but further research is needed. [28] The proteasome is a cellular complex that is responsible for breaking down damaged proteins into their constituent amino acids. As individuals age, the functionality of the proteasome may decline, which can result in the accumulation of damaged proteins within cells. This accumulation of damaged proteins can contribute to cellular dysfunction and the development of age-related diseases. Therefore, there is growing interest in exploring ways to activate the proteasome or enhance its function as a potential strategy for promoting healthy aging. [29] Dysregulation of Cyclin D1 expression or activity as observed in healthy elderly could disrupt the normal cell cycle in BM-HSCs. This disruption might trigger apoptosis pathways, leading to increased stem cell death. The degradation of AXIN and DVL in BM-HSCs of healthy elderly could impact the Wnt signaling pathway which is dysregulated in aging. [30] A negative enrichment was observed in lymphocyte-mediated immunity, RNA splicing, negative regulation of cytokine production, response to leukemia inhibitory factor (LIF), Bone Morphogenetic Protein (BMP), adaptive immune response, lymphocyte activation, stem cell proliferation, and protein deacetylation in BM-HSCs of elderly. The ability of immune cells to regulate cytokine production, efficiency of lymphocyte activation and reduced stem cell proliferation leading to decreased production of naive lymphocytes and a less robust immune system in the elderly is known. [31] [32] Other processes, such as RNA splicing and protein deacetylation, were dysregulated with age, which can adversely impact the humoral immune system. [31, 33,34] BMP signaling was negatively enriched in the BM-HSCs of elderly and is essential for the maintenance and self-renewal of adult HSCs, as well as for promoting their self-renewal and repopulating potential of adult HSCs. [34] [35]

Proteins that bind to telomeres are crucial for maintaining telomere integrity and preventing excessive shortening. A disruption in telomeric binding proteins, as observed in BM-HSCs of elderly individuals, could lead to accelerated telomere shortening, resulting in cellular dysfunction. [36] Similarly, changes in nucleoside binding proteins with age could impact RNA processing and potentially result in problems with gene expression and protein production. [34] SMAD4, a protein involved in the transforming growth factor-beta (TGF-β) signaling pathway, which was negatively enriched in the BM-HSCs of elderly individuals, and the dysregulation of TGF-β signaling can potentially contribute to age-related pathologies. [35] Glycosaminoglycans (GAGs) are complex sugar molecules that form part of the extracellular matrix and can bind to various proteins. Alterations in GAG composition or the proteins that bind GAGs with age could contribute to impaired BM-HSCs function. [37] Additionally, negative enrichment of DNA demethylase activity in the BM-HSCs of elderly individuals may also play a role in aging-related processes such as cellular dysfunction, impaired DNA repair, increased inflammation, and disrupted gene expression.[38] However, further research is necessary to fully understand how these pathways and processes can be targeted to promote healthy aging in humans. Some of the drugs or natural products such as Salermide, Celestrol, Cercosporin dichloride, Dorsomorphin monohydrochloride, and LDN-193189 monohydrochloride were found to reverse the aging associated gene signatures. Salermide is a small molecule that has been shown to activate sirtuins, a class of proteins involved in cellular stress response and longevity pathways. Sirtuins are thought to promote healthy aging by regulating various processes like metabolism, inflammation, and DNA repair. [39] More research is needed to confirm the effects of salermide on sirtuins and its potential benefits for aging in humans. Celestrol is a leptin sensitizer and a naturally occurring compound found in the Tripterygium wilfordii plant.[40] Studies suggest it has anti-inflammatory, anti-aging, and neuroprotective properties [40] [41]. More research is needed to determine the safety and efficacy of celestrol for promoting healthy aging in humans. Cercosporin is a metabolite of the fungus Cercospora species which is a source of anti-aging polyketides targeting 26S proteasome. [42] Dorsomorphin and LDN-193189 have demonstrated the ability to inhibit BMP-mediated Smad, p38, and Akt signaling, which may contribute to modulating anti-aging effects. [43] Further research is necessary to confirm the anti-aging potential of the drugs and natural products identified in this study. In vitro and in vivo model systems should be used to validate the results before conducting preclinical evaluations.

## Conclusion:

This study utilized RNA-seq data from BM-HSCs of healthy elderly and young individuals to investigate the cellular and molecular mechanisms associated with aging. The findings provide insights into the potential age-related changes in cellular and molecular pathways and GOs that significantly contribute to BM-HSCs dysfunction. By understanding these molecular alterations, we can pave the way for future research aimed at developing strategies to maintain HSC function and regenerative capacity in the elderly population and potentially extend the healthspan by targeting mechanisms that contribute to BM-HSCs decline with age. Further in-depth studies are warranted to validate the drugs that reverse the BM-HSCs aging associated gene signatures and translate them into potential therapeutic interventions. Hence, by unraveling the molecular landscape of aging BM-HSCs, we can move closer to the goal of promoting the healthspan and improving the lives of elderly individuals.

## Conflict of interest statement:

The author hereby affirms that no connections or involvement with any organization or entity that possesses a financial stake in the topics or materials presented in this manuscript.

## Author Contribution Statement:

Conceptualization: HK; Methods and Data Analysis: HK; data curation and formal analysis: HK; writing-original draft, review and editing: HK. HK read and consented to the final version of the manuscript.

## Figures and Tables

**Table 1:** The top 25 drugs or natural products that reverse DEGs in the BM-HSCs of healthy old on L1000CDS2 analysis

**Rank**	**score**	**Drug or Natural Product (Perturbation)**	**Cell-line**	**Dose**	**Time**
1	0.0462	Salermide	PC3	120.0um	24.0h
2	0.0443	BRD-K92317137	VCAP	10.0um	6.0h
3	0.0443	F1566-0341	MCF7	10.0um	6.0h
4	0.0425	Dorsomorphin dihydrochloride	MCF7	10.0um	24.0h
5	0.0406	BRD-K92317137	A549	10.0um	6.0h
6	0.0406	PK-11195	HT29	160.0um	24.0h
7	0.0397	Chemistry 2804	HT29	10.0um	24.0h
8	0.0397	Celastrol	BT20	1.11um	24h
9	0.0388	MLN2238	MCF7	10.0um	24.0h
10	0.0388	TW 37	VCAP	10.0um	24.0h
11	0.0369	BRD-A18763547	HT29	10.0um	24.0h
12	0.0369	BRD-K94325918	MCF7	10.0um	24.0h
13	0.036	manumycin A	PC3	10.0um	24.0h
14	0.036	BRD-K17140735	HT29	11.1um	6.0h
15	0.036	BRD-K23478508	MCF7	10.0um	24.0h
16	0.0351	ARP 101	MCF7	10.0um	24.0h
17	0.0351	MLN2238	PC3	10.0um	24.0h
18	0.0351	BRD-K91370081	HT29	10.0um	6.0h
19	0.0332	Cercosporin	HT29	10.0um	24.0h
20	0.0332	BRD-A62809825	HT29	10.0um	24.0h
21	0.0332	BRD-K43620258	MCF7	80.0um	24.0h
22	0.0332	TW 37	PC3	10.0um	24.0h
23	0.0332	BRD-K32896438	MCF7	10.0um	24.0h
24	0.0332	LDN-193189	HT29	10um	24h
25	0.0323	Puromycin dihydrochloride	HA1E	10.0um	6.0h
